# Changes in microbial community phylogeny and metabolic activity along the water column uncouple at near sediment aphotic layers in fjords

**DOI:** 10.1038/s41598-021-98519-2

**Published:** 2021-09-29

**Authors:** Sven P. Tobias-Hünefeldt, Stephen R. Wing, Federico Baltar, Sergio E. Morales

**Affiliations:** 1 Department of Microbiology and Immunology, University of Otago, 720 Cumberland Street, North Dunedin, PO Box 56, Dunedin, 9054 New Zealand; 2 Department of Experimental Limnology, Leibniz-Institude of Freshwater Ecology and Inland Fisheries (IGB), 16775 Neuglobsow, Germany; 3 Department of Marine Science, University of Otago, PO Box 56, Dunedin, 9054 New Zealand; 4 Department of Functional and Evolutionary Ecology, University of Vienna, Althanstrasse 14, 1090 Vienna, Austria

**Keywords:** Molecular ecology, Microbial ecology

## Abstract

Fjords are semi-enclosed marine systems with unique physical conditions that influence microbial community composition and structure. Pronounced organic matter and physical condition gradients within fjords provide a natural laboratory for the study of changes in microbial community structure and metabolic potential in response to environmental conditions. Photosynthetic production in euphotic zones sustains deeper aphotic microbial activity via organic matter sinking, augmented by large terrestrial inputs. Previous studies do not consider both prokaryotic and eukaryotic communities when linking metabolic potential and activity, community composition, and environmental gradients. To address this gap we profiled microbial functional potential (Biolog Ecoplates), bacterial abundance, heterotrophic production (^3^H-Leucine incorporation), and prokaryotic/eukaryotic community composition (16S and 18S rRNA amplicon gene sequencing). Similar factors shaped metabolic potential, activity and community (prokaryotic and eukaryotic) composition across surface/near surface sites. However, increased metabolic diversity at near bottom (aphotic) sites reflected an organic matter influence from sediments. Photosynthetically produced particulate organic matter shaped the upper water column community composition and metabolic potential. In contrast, microbial activity at deeper aphotic waters were strongly influenced by other organic matter input than sinking marine snow (e.g. sediment resuspension of benthic organic matter, remineralisation of terrestrially derived organic matter, etc.), severing the link between community structure and metabolic potential. Taken together, different organic matter sources shape microbial activity, but not community composition, in New Zealand fjords.

## Introduction

Fjords are unique environments, representing modified marine ecosystems mixing freshwater, terrestrial and marine inputs. Influences on microbial community structure and function are linked to changes in environmental condition, including alternate organic carbon sources (e.g. terrestrial, marine and freshwater sources), salinity, nutrient, and light^1–3^. Moreover, due to these strong environmental gradients, fjords are ideal natural laboratories to study marine microbial communities and phylogenetic and functional diversity controls due to strong environmental gradients. However, the energy sources supporting primary production and heterotrophic activity in fjords, and how they change in relation to observed community changes, remain poorly defined. In open ocean systems primary productivity by surface phytoplankton mediates the downward flux of particulate carbon, transferring energy to aphotic zones. This unidirectional transfer of organic matter from surface to deeper layers is termed the biological carbon pump^4–6^. The process is expected to dominate in fjords where carbon inputs are predominantly linked to phytoplankton and chemoautotroph production^7–9^, sustaining a significant portion of heterotrophic respiration^10^. Nevertheless, studies of benthic communities in fjords have demonstrated that microbial reworking of refractory organic matter from terrestrial sources is in some fjords a dominant source of carbon to deep communities^11–14^. Despite this, we lack an integrated view of microbial metabolic potential within fjords and specific information about the composition of microbial populations and how they are linked to the available range of organic matter sources. Resolution of these associations will provide the basis for a mechanistic understanding of how organic matter is processed in fjords and increased understanding of how this ecosystem is sustained and shaped.

In a recent study we examined for the first time the patterns in microbial community composition relative to variability in environmental factors among fjords in the New Zealand Fiordland system^15^. This previous study identified that salinity and depth were the primary drivers of both prokaryotic and eukaryotic microbiome diversity and composition changes, while oxygen and temperature only played a minor role in determining taxonomic patterns. However, other factors such as tannin induced light penetration decreases or grazing pressures may also play a role^16,17^, especially when there are differences between prokaryotic and eukaryotic community patterns. The depth and salinity effects are shown to be linked to primarily the LSL (low-salinity layer), with distinct diversity and community differences between Chalky Inlet (containing the smallest LSL) and Doubtful Sound (containing the largest LSL). Microbial phyla matched previous reports in marine, and tropical and tundra fjords. However, the identified phyla are capable of diverse functional processes and the link between patterns in phylogenetic and functional diversity in these fjords remained unresolved.

In the present study we examine the microbial metabolic community potential (via Biolog Ecoplates), bacterial abundance, heterotrophic production (via ^3^H-leucine incorporation), and prokaryotic/eukaryotic community composition (via 16S and 18S rRNA amplicon gene sequencing). We use this data to compare community metabolic diversity and potential, and how it relates to known drivers of microbial community changes across six different fjords in New Zealand. We aimed to reveal how metabolic potential changed along environmental gradients within fjords and how these patterns are linked to community composition changes, integrating both prokaryotic and eukaryotic microbiomes for the first time. We hypothesised that microbial community function and composition were linked, and both would decrease with depth due to decreased abundance of photosynthetically produced organic matter.

## Methods

### Sample collection, DNA extraction, and sequencing

Samples were collected and processed according to the protocol outlined in Tobias-Hünefeldt et al.^15^. Duplicate samples were collected from Fiordland National Park (45.60° S, 167.36° E) in November 2015 using a CTD sensor system (SBE-25 0352) with attached 10 L Niskin bottles for a total of 44 samples. Samples were collected at the head and mouth of 5 fjords (Breaksea Sound, Chalky Inlet, Doubtful Sound, Dusky Sound, and Wet Jacket Arm) at the surface and at 10 m. However, 1, 1, 4, 1, and 4 samples were collected from Breaksea Sound’s 10 m mouth, Chalky Inlet's 10 m head, Doubtful Sound's surface head, Dusky Sound's 10 m head, and Wet Jacket Arm's surface head region. To generate higher resolution data sets Long Sound samples were collected using a transect and depth profile. Duplicate surface and 10 m samples from the transect started at the fjord's mouth and moving inwards with sampling occurring at 2.47, 3.16, 4.73, 5.59, 8.47, 10.67, and 14.3 km from the outermost sample with exceptions. The depth profile was collected using duplicate samples 8.47 km from the outermost sample at depths of 0, 10, 40, 100, 200, and 300 m, with exceptions.

DNA was extracted using the MoBio DNeasy^®^ PowerSoil^®^ Kit (MoBio, Solana Beach, CA, USA) using the filter contents of 0.22 µm polycarbonate (diameter of 47 mm) filtered 0.5–1 L water subsample, frozen at − 20 °C and stored at − 80 °C. A modified manufacturers protocol was utilised, bead beating samples in a Geno/Grinder for 2 × 15 s instead of vortexing at maximum speed for 10 min.

DNA underwent sequencing and quality control as outlined in Tobias-Hünefeldt et al.^15^. The Earth Microbiome Project protocol was followed^18^, generating community profiles with barcoded 16S (targeting the V4 region: 515F (5′-NNNNNNNNGTGTGCCAGCMGCCGCGGTAA-3′) and 806R (5′-GGACTACHVGGGTWTCTAAT-3′)) or 18S 1391f (5′-GTACACACCGCCCGTC-3′) and EukBr (5′-TGATCCTTCTGCAGGTTCACCTAC-3′) rRNA gene primers. Illumina HiSeq (16S) and MiSeq (18S) 2 × 151 bp runs were used to generate community reads. Raw community profiles underwent quality control using the Quantitative Insights into Molecular Ecology (QIIME) 1.9.1 open-reference operation taxonomic unit-picking workflow with default parameters^19^. Operational taxonomic units (OTUs) were clustered at 97% for 16S and 99% for 18S similarity using UCLUST^20^ and an open reference strategy based on reference sequences from the SILVA database (release 128) using QIIME’s pick_open_reference_otus.py command^21^. OTUs were classified to 7 taxa levels (kingdom, phylum, class, order, family, genus, OTU) using BLAST^22^ with a maximum e-value of 0.001 against the SILVA database. Subsampling and rarefication was carried out ten times at a depth of 22,000 (16S) and 6600 (18S) sequences, merging the resulting tables into a single OUT table. The merged OTU table was then exported as a biom (json) file for further processing.

All sequence data from this study has been deposited in NCBI under BioProject PRJNA540153.

All data analysis was carried out using R version 3.6.1 (R Core Team (2019). R: A language and environment for statistical computing. R Foundation for Statistical Computing, Vienna, Austria. URL https://www.R-project.org/) within RStudio^23^, and visualised using the ggplot2 package (version 3.2.1)^24^ unless otherwise stated. All code and associated files are available at https://github.com/SvenTobias-Hunefeldt/Fiordland_2021/.

### Carbon utilisation profiling, and bacterial abundance and productivity

Carbon utilisation profiles were determined using Biolog EcoPlates™ loaded with water collected in 10 L Niskin bottles attached to a CTD rosette.  150 μL of sample was utilised per Biolog Ecoplate well, then incubated for 7 days at 4 °C with colour patterns assessed at OD A590 nm.

Samples were incubated in the dark for 10 min, then preserved with glutaraldehyde (final concentration of 2%) and frozen with liquid nitrogen prior to counting. Thawed cells were stained with a final concentration of 10 µL ml^−1^ SYBR Green I fluorescent staining for 10 min, bacterial abundance quantifications were performed with a FACS Canto II flow cytometer (Benton and Dickinson, USA) following methods in Gasol and Del Giorgio^25^.

Leucine incorporation assays were used to quantify heterotrophic bacterial productivity, following the centrifugation protocol outlined in Smith and Azam^26^. Triplicate 1.2 mL samples received a saturating concentration (40 nmol l^−1^) of ^3^H-Leucine (Perkin–Elmer, specific activity = 169 Ci mmol^−1^). The addition of 120 μL of 50% trichloroacetic acid (TCA) 10 min prior to isotope addition established controls. Microcentrifuge tubes were incubated in the dark at in situ temperature for 1 h. Leucine incorporation in triplicate samples was stopped with the addition of 120 μL ice-cold 50% TCA. Subsamples and controls were kept at − 20 °C until centrifugation (at ca. 12,000*g*) for 20 min, followed by aspiration. Finally, 1 mL of scintillation cocktail was added to the microcentrifuge tubes before determining the incorporated radioactivity after 24–48 h on a Tri-Carb^®^ Liquid Scintillation Counters scintillation counter (Perkin–Elmer) with quenching correction.

### Statistical analyses

Beta-diversity was explored using the Stats package, and displayed with the ggplot2, ggpubr, and ggbiplot packages ^24,27,28^. Findings were corroborated with phyloseq package generated NMDS plots. Dissimilarities were calculated with the vegan package vegdist() function using Bray-Curtis distances ^29^. Vegan package (version 2.5-6) mantel tests, ANOSIM, and PERMANOVA tests assessed multi-group correlations, while Wilcoxon tests were used to compare two groups.

## Results and discussion

**Figure 1:**
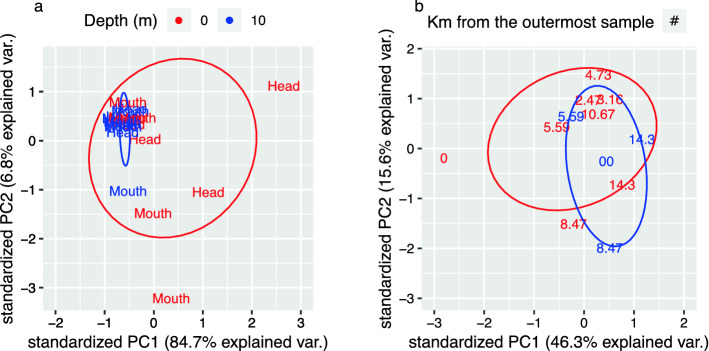
Comparison of Biolog Ecoplates results for surface vs. 10m samples by principal component analysis (PCA). Surface and 10m samples compared across 5 sites. (**a**) Comparison of samples from a transect in a single site (Long Sound). (**b**) Text labels represent horizontal sample location (head/mouth of the fjord [**a**], or Km from the outermost sample [**b**]). Ellipses represent the 95% confidence interval.

**Figure 2:**
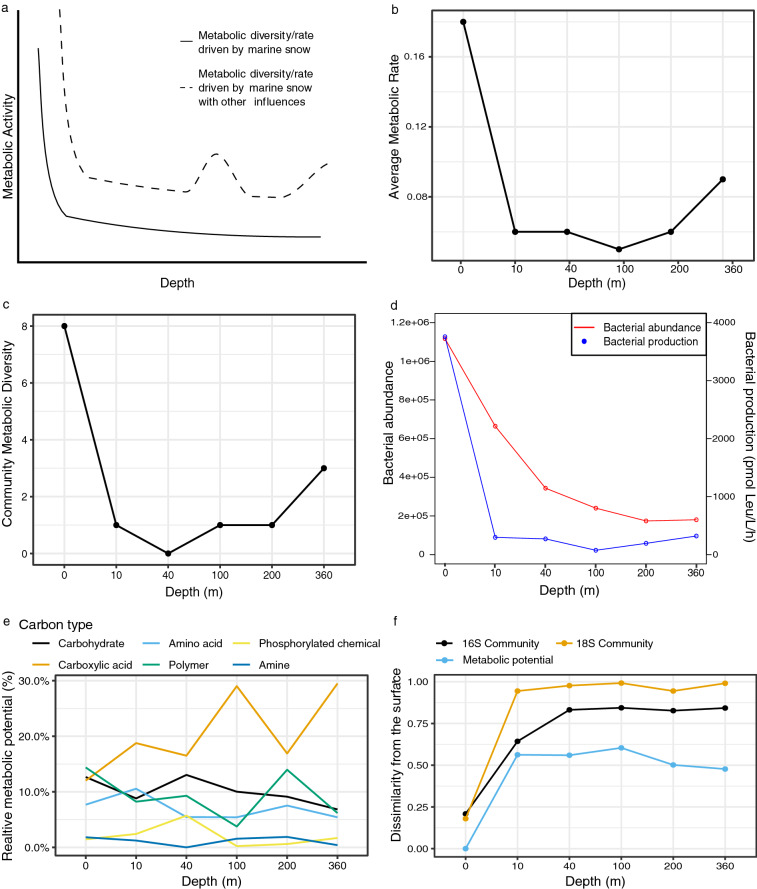
Benthic and surface influence on metabolic potential. Two potential metabolic scenarios are depicted (**a**), the metabolic rate and diversity when driven solely by photosynthetic production, and another model that accounts for additional benthic influences. Biolog Ecoplate plate derived Average Metabolic Rate (AMR, **b**), Community Metabolic Diversity (**c**), and the relative metabolic potential (**e**) are also shown in addition to the bacterial abundance and productivity (**d**), and taxonomic and Biolog plate derived dissimilarity (Bray–Curtis) from the surface (**f**). Different colours represent carbon source groups (**e**; carbohydrates are blue, carboxylic acids are orange, amino acids are light blue, polymers are green, phosphorylated chemicals are yellow, and amines are dark blue), and Bray–Curtis dissimilarity data sources (**f**; the 16S community is black, 18S community is orange, and Biolog derived metabolic potential is light blue).

The present study was carried out in six fjords within New Zealand’s Fiordland system, specifically Breaksea Sound, Chalky Inlet, Doubtful Sound, Dusky Sound, Long Sound, and Wet Jacket Arm, as described in Tobias-Hünefeldt et al.^15^. Analyses were divided into three categories: (1) a multi-fjord analysis comprising five of the tested fjords (excluding Long Sound), (2) a high-resolution study along Long Sound’s horizontal axis, and (3) a depth profile of Long Sound’s deepest location (at 421 m). These categories were established to identify trends across multiple fjords, and then test the trends using a fjord analysed at a higher resolution. Total community composition (via 16S and 18S rRNA gene sequencing) and metabolic potential did not significantly covary across the five studied fjords (Mantel, r < 0.01, P = 0.47), Long Sound’s horizontal transect (Mantel, r < 0.01, P > 0.05) (Fig. 1), or Long Sound’s depth profile (Mantel, r < 0.22, P > 0.05) (Fig. 2). However, depth covaried with community structure for five studied fjords (Fig. S1), across the horizontal transect at Long Sound (Figs. S2, S3), and along Long Sound’s depth profile (Fig. 2). Microbial communities differed significantly between the surface and 10 m (Mantel, Multi-fjord—r = 0.21, P < 0.01, Transect—prokaryotes r = 0.47, P ≤ 0.01, eukaryotes r = 0.56, P < 0.01), as opposed to changes along the fjords horizontal axis (Mantel, Multi-fjord—r = 0.08, P = 0.04, Transect—prokaryotes r = 0.21, P = 0.01, eukaryotes r = 0.13, P = 0.07) (Figs. S2, S3, S4). Significant depth dependent metabolic potential differences could also be identified at the regional (multi-fjord; Anosim: R = 0.10, P = 0.03) and individual fjord scale ﻿(Anosim: R = 0.27, P ≤ 0.01) (Fig. 1).

Across the fjords (excluding Long Sound), surface samples were more metabolically active (i.e., average metabolic rate [AMR]) compared to the 10 m samples (Wilcox test, W = 425, P < 0.01), and samples closer to the fjord head displayed increased metabolic rates (Wilcox test, W = 0, P < 0.01). Thus, metabolic variability varied with horizontal sampling location (Fig. 1b). While activity was not consistent along the length of Long Sound, surface samples in the low salinity layer were more metabolically active than those collected at 10 m (Fig. S5), although activity at 10 m between 1 and 4 km could not be measured due to sampling limitations. Heterotrophic production (via leucine incorporation) was not significantly correlated with microbial abundance within the five studied fjords and Long Sounds horizontal axis (Mantel—Multi-fjord r = 0.04, P = 0.22, Horizontal r = 0.04, P = 0.32). Along the depth profile, prokaryotic abundance and production were significantly correlated (Mantel, r = 0.60, P = 0.01) exhibiting a large drop in productivity from the surface to 10 m followed by a more gradual decrease.

We hypothesized that metabolic rate and diversity would be driven by marine snow linked to photosynthetic primary producers at the surface (e.g. phytoplankton and macroalgae; Fig. 2a) leading to a steady decrease in metabolic potential as resources were depleted with increases in depth. The high-resolution depth profile was used to explore this topic in more detail (Fig. 2, Fig. S4). Any deviation altering the slow loss of metabolic potential would be linked to extraneous sources of nutrients uncoupled from surface activity (i.e. benthic influences, subsidies from land-based inputs). We observed a steady loss of metabolic diversity and rate from surface to 40 m (Fig. 2b,c), with sustained increases at depths from 100 m onwards. However, abundance did not follow the same pattern, and instead continuously decreased until 360 m (Fig. 2d). Abundance and metabolic changes over depth were associated with shifts in specific carbon utilization potential, where carbohydrate metabolism decreased from 12.7 to 6.8%, as carboxylic acid utilization increased from 12.0 to 29.5% from the surface to 360 m (Fig. 2e). This likely reflected the diminishing abundance of readily mineralizable substrates with depth, and the increase in recalcitrant sources of carbon and energy. Consistently, we also observed increases in phosphorylated chemical metabolism peaking at 40 and 360 m (Fig. 2e) as expected from utilization of phosphorous at the surface during blooms^30^. However, observed changes in metabolic potential did not reflect changes in prokaryotic or eukaryotic community composition (Fig. 2, Fig. S4), suggesting that while the community members were relatively consistent past a certain depth (i.e., 10 m for eukaryotes and 40 m for prokaryotes) the metabolic potential changed dynamically past 100 m, regaining peak metabolic potential with proximity to the bottom (Fig. 2f). Therefore, we conclude that as bacterial abundance decreases with depth, the functional diversity for carbon utilisation increases. However, this is not due to large prokaryotic or eukaryotic community changes, but may be due to the utilisation of alternative metabolic pathways by the present organisms. However, it may still be possible that fungi play a role in the observed metabolic changes as this study did not assess their community composition. This study is nonetheless one of the first to consider both prokaryotic and eukaryotic community compositions when assessing metabolic changes within a fjord.

Our results demonstrate that metabolic potential and activity in fjords is linked to similar parameters as microbial community composition across surface or near surface sites. However, distinct selective pressures exist at aphotic sites which ultimately affect the link between phylogenetic and metabolic diversity. The observed patterns are contrary to the open ocean carbon pump paradigm and demonstrate that additional refractory sources of organic matter, including resuspension of terrestrial organic matter associated with benthic communities, are important contributors to microbial activity in fjords, which form a major marine biome worldwide (e.g. Patagonian, Scandinavian, Northeastern Pacific systems). We propose that this reflects the influence of the benthic microbial loop and incorporation and breakdown of terrestrial organic matter in fjordic sediments. Sediment resuspension can occur through a variety of abiotic^31,32^ and biotic sources (known as bioturbation^33^). The resuspension of organically rich sediments has previously been shown to increase microbial activity^34^. Observed patterns suggest that resuspension could also be driven by bottom feeding organisms, increasing suspended organic matter and its utilization in near bottom habitats^35^. Therefore, organic matter sources influence the relationship between microbial communities and their metabolic potential.

## Supplementary Information

Supplementary Legends.

Supplementary Figure S1.

Supplementary Figure S2.

Supplementary Figure S3.

Supplementary Figure S4.

Supplementary Figure S5.

Supplementary Table S1.

## Data Availability

The sequence data from this study have been deposited in NCBI under BioProject PRJNA540153. All data generated and/or analysed during the study is available within the GitHub repository, https://github.com/SvenTobias-Hunefeldt/Fiordland_2021/.

## References

[1] Mckee D, Cunningham A, Jones KJ (2002). Optical and hydrographic consequences of freshwater run-off during spring phytoplankton growth in a Scottish fjord. J. Plankton Res..

[2] Pulchan JK, Helleur R, Abrajano TA (2003). TMAH thermochemolysis characterization of marine sedimentary organic matter in a Newfoundland fjord. Organic Geochemistry.

[3] Cui X, Bianchi TS, Savage C, Smith RW (2016). Organic carbon burial in fjords: Terrestrial versus marine inputs. Earth Planet. Sci. Lett..

[4] Jiao N (2010). Microbial production of recalcitrant dissolved organic matter: Long-term carbon storage in the global ocean. Nat. Rev. Microbiol..

[5] Jiao N, Zheng Q (2011). The microbial carbon pump: From genes to ecosystems. Appl. Environ. Microbiol..

[6] Legendre L, Rivkin RB, Weinbauer MG, Guidi L, Uitz J (2015). The microbial carbon pump concept: Potential biogeochemical significance in the globally changing ocean. Prog. Oceanogr..

[7] Amy PS, Caldwell BA, Soeldner AH, Morita RY, Albright LJ (1987). Microbial activity and ultrastructure of mineral-based marine snow from Howe Sound, British Columbia. Can. J. Fish. Aquat. Sci..

[8] Albright LJ, McCrae SK, May BE (1986). Attached and free-floating bacterioplankton in Howe Sound, British Columbia, a coastal marine fjord-embayment. Appl. Environ. Microbiol..

[9] Alldredge AL (2002). Occurrence and mechanisms of formation of a dramatic thin layer of marine snow in a shallow Pacific fjord. Mar. Ecol. Prog. Ser..

[10] Iturriaga R, Hoppe HG (1977). Observations of heterotrophic activity on photoassimilated organic matter. Mar. Biol..

[11] McLeod RJ, Wing SR, Skilton JE (2010). High incidence of invertebrate-chemoautotroph symbioses in benthic communities of the New Zealand fjords. Limnol. Oceanogr..

[12] Jack L, Wing SR, McLeod RJ (2009). Prey base shifts in red rock lobster *Jasus edwardsii* in response to habitat conversion in Fiordland marine reserves: Implications for effective spatial management. Mar. Ecol. Prog. Ser..

[13] McLeod RJ, Wing SR (2009). Strong pathways for incorporation of terrestrially derived organic matter into benthic communities. Estuar. Coast. Shelf Sci..

[14] McLeod RJ, Wing SR (2007). Hagfish in the New Zealand fjords are supported by chemoautotrophy of forest carbon. Ecology.

[15] Tobias-Hünefeldt SP, Wing SR, Espinel-Velasco N, Baltar F, Morales SE (2019). Depth and location influence prokaryotic and eukaryotic microbial community structure in New Zealand fjords. Sci. Total Environ..

[16] Barrett NS, Edgar GJ (2010). Distribution of benthic communities in the fjord-like Bathurst Channel ecosystem, south-western Tasmania, a globally anomalous estuarine protected area. Aquat. Conserv. Mar. Freshw. Ecosyst..

[17] Copeland A (2012). Geomorphic features and benthic habitats of a Sub-Arctic Fjord: Gilbert Bay, Southern Labrador, Canada. Seafloor Geomorphol. Benthic Habitat..

[18] Caporaso JG (2012). Ultra-high-throughput microbial community analysis on the Illumina HiSeq and MiSeq platforms. ISME J..

[19] Caporaso JG (2010). correspondence QIIME allows analysis of high- throughput community sequencing data Intensity normalization improves color calling in SOLiD sequencing. Nat. Publ. Gr..

[20] Edgar RC (2010). Search and clustering orders of magnitude faster than BLAST. Bioinformatics.

[21] Quast C (2013). The SILVA ribosomal RNA gene database project: Improved data processing and web-based tools. Nucleic Acids Res..

[22] Altschul SF (1997). Gapped BLAST and PSI-BLAST: A new generation of protein database search programs. Nucleic Acids Res..

[23] R Core Team. R: A Language and Environment for Statistical Computing. R Foundation for Statistical Computing, Vienna. (2018).

[24] Wickham H (2016). ggplot2: Elegant Graphics for Data Analysis.

[25] Gasol JM, Del Giorgio PA (2000). Using flow cytometry for counting natural planktonic bacteria and understanding the structure of planktonic bacterial communities. Scientia Marina.

[26] Smith DC, Azam F (1992). A simple, economical method for measuring bacterial protein synthesis rates in seawater using 3H-leucine 1. Mar. Microb. Food Webs.

[27] Kassambara, A. ggpubr: "ggplot2" based publication ready plots. (2019).

[28] Vincent, Q.V. ggbiplot: A ggplot2 based biplot. (2011).

[29] Oksanen, J. et al. vegan: Community Ecology Package. (2019).

[30] Tiselius P, Kuylenstierna M (1996). Growth and decline of a diatom spring bloom: Phytoplankton species composition, formation of marine snow and the role of heterotrophic dinoflagellates. J. Plankton Res..

[31] Pickrill RA (1987). Circulation and sedimentation of suspended particulate matter in New Zealand fjords. Mar. Geol..

[32] Christiansen C, Zacharias I, Vang T (1992). Storage, redistribution and net export of dissolved and sediment-bound nutrients, Vejle Fjord, Denmark. Hydrobiologia.

[33] Mevenkamp L (2017). Impaired short-term functioning of a benthic community from a deep Norwegian fjord following deposition of mine tailings and sediments. Front. Mar. Sci..

[34] Flindt MR, Kamp-Nielsen L (1998). The influence of sediment resuspension on nutrient metabolism in the eutrophic Roskilde Fjord, Denmark. SIL Proc..

[35] Yahel G (2008). Fish activity: A major mechanism for sediment resuspension and organic matter remineralization in coastal marine sediments. Mar. Ecol. Prog. Ser..

