# Supplementary material for: Changes in microbial community phylogeny and metabolic activity along the water column uncouple at near sediment aphotic layers in fjords
Source: Sci Rep. 2021 Sep 29;11:19303. doi: 10.1038/s41598-021-98519-2 (PMC8481465; doi:10.1038/s41598-021-98519-2)
Supplement: Supplementary file 1 — Supplementary Legends. [file 41598_2021_98519_MOESM1_ESM.docx]

**Supplementary Figure Legends**

**Figure S1.** **Microbial beta-diversity of five fjords.** The fjord of origin, sample region, and depth were used to identify sample origin. Dissimilarity was assessed using Bray-Curtis distance matrices based on OTUs at 97% similarity. Ellipses represent the 95% confidence intervals of different sample depths, while shapes represent sample origin, both regional (inner = hollow, outer = filled), and depth (surface = triangles, 10 m = squares).

**Figure S2.** **Prokaryotic beta-diversity across Long Sound.** Beta-diversity based on 16S gene data for Long Sound’s horizontal axis. Dissimilarity was assessed using Bray-Curtis distance matrices based on OTUs at 97% similarity.

**Figure S3.** **Eukaryotic beta-diversity across Long Sound.** Beta-diversity based 18S data for Long Sound’s horizontal axis. Dissimilarity was assessed using Bray-Curtis distance matrices based on OTUs at 97% similarity.

**Figure S4. Community structure of Long Sounds depth profile**. The mean relative abundance of phyla is shown for Long Sound’s depth profile, based on 16S (a) and 18S (b) sequencing. Phyla below 1% relative abundance have been grouped into Rare Taxa (<1%). Error bars represent mean abundance standard error from duplicate samples.

**Figure S5. The average metabolic rate across Long Sound.** The average metabolic rate (AMR) across Long Sound’s horizontal axis, different colours represent different depth (red represents surface samples and blue 10 m).

**Table S1. Statistical results for ANOSIM and ADONIS test.**
