# Supplementary figures and images for: Changes in microbial community phylogeny and metabolic activity along the water column uncouple at near sediment aphotic layers in fjords
Source: Sci Rep. 2021 Sep 29;11:19303. doi: 10.1038/s41598-021-98519-2 (PMC8481465; doi:10.1038/s41598-021-98519-2)

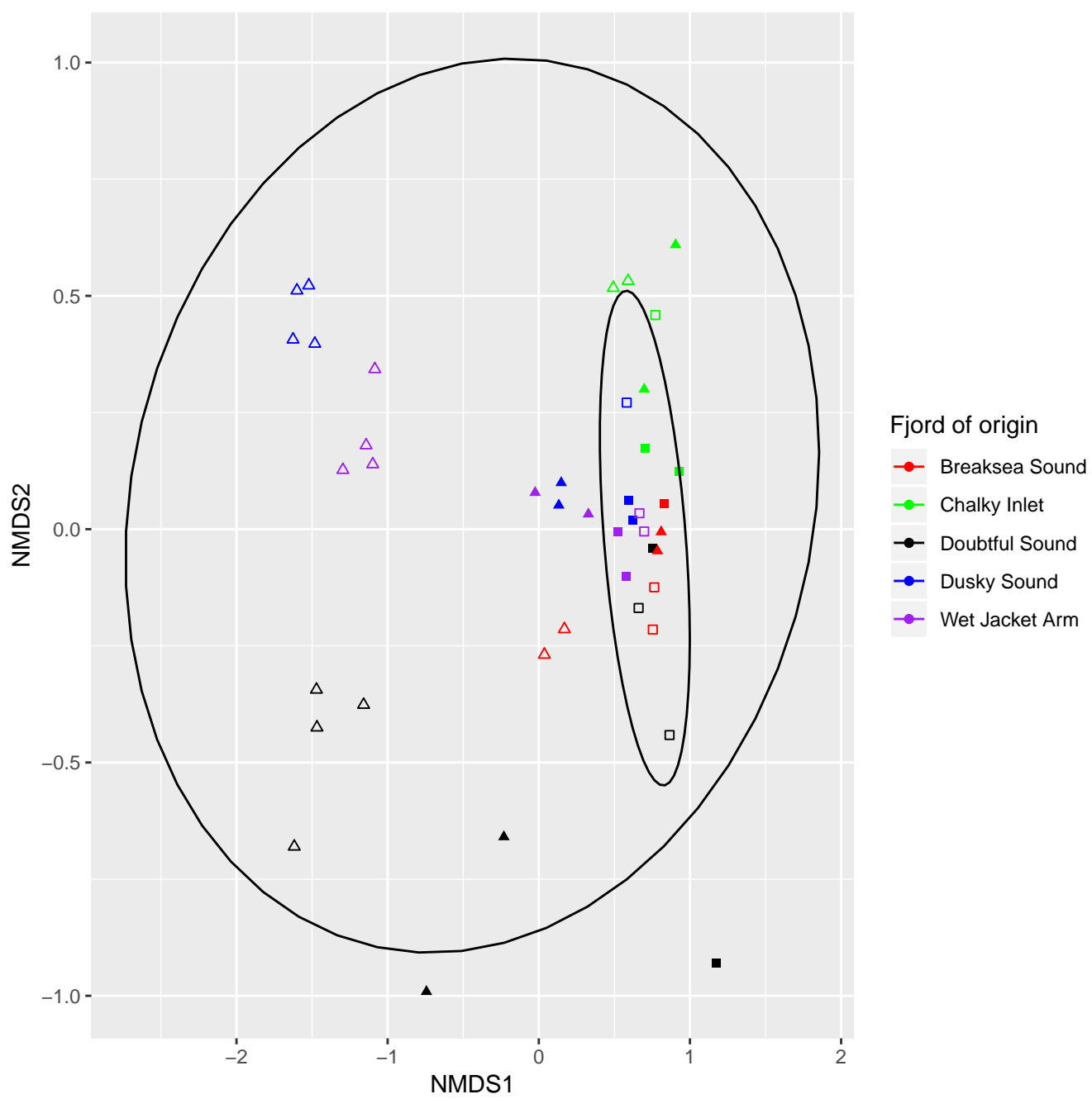

Supplement: Supplementary file 2 — Supplementary Figure S1. [file 41598_2021_98519_MOESM2_ESM.pdf]

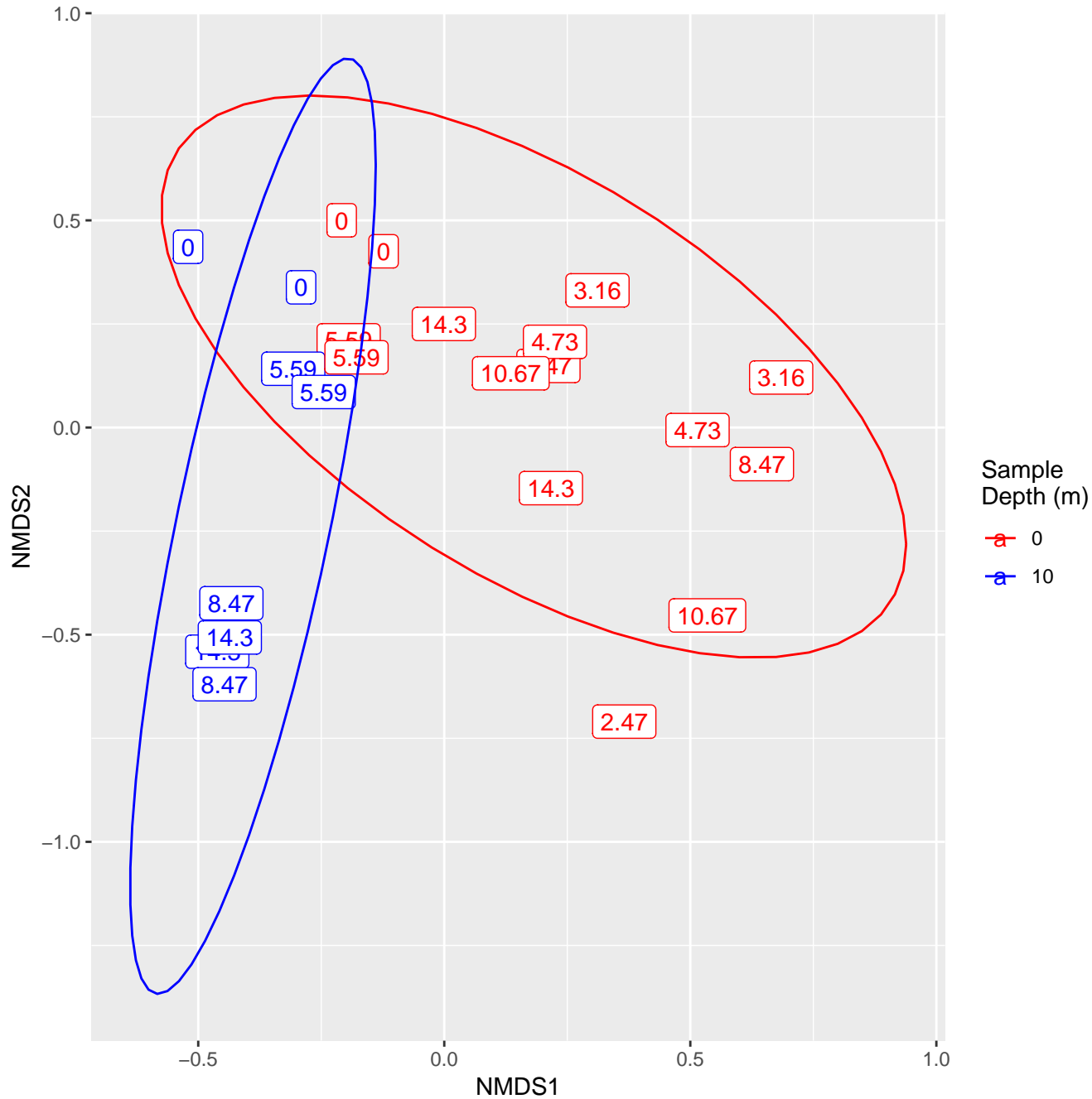

Supplement: Supplementary file 3 — Supplementary Figure S2. [file 41598_2021_98519_MOESM3_ESM.pdf]

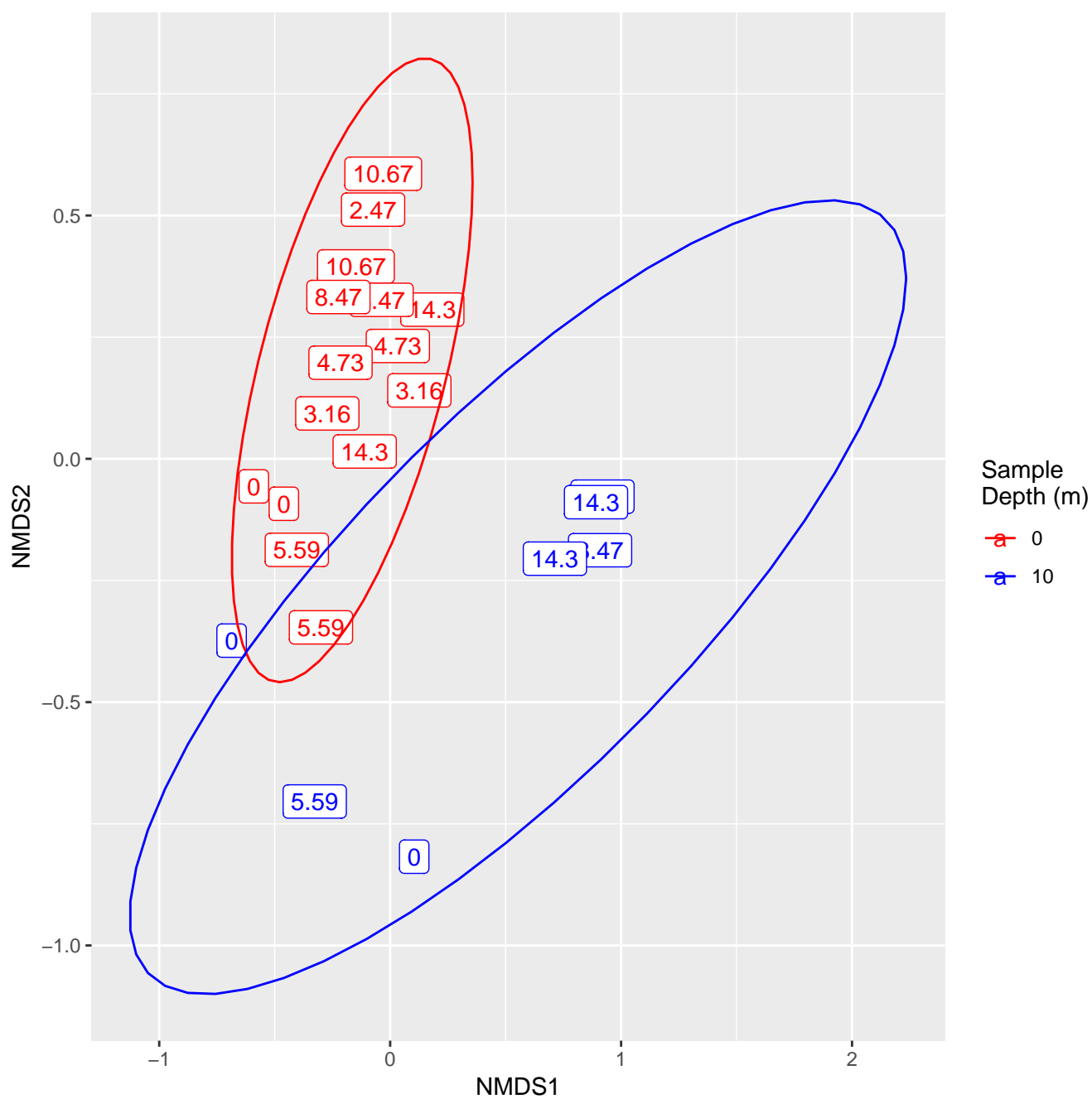

Supplement: Supplementary file 4 — Supplementary Figure S3. [file 41598_2021_98519_MOESM4_ESM.pdf]

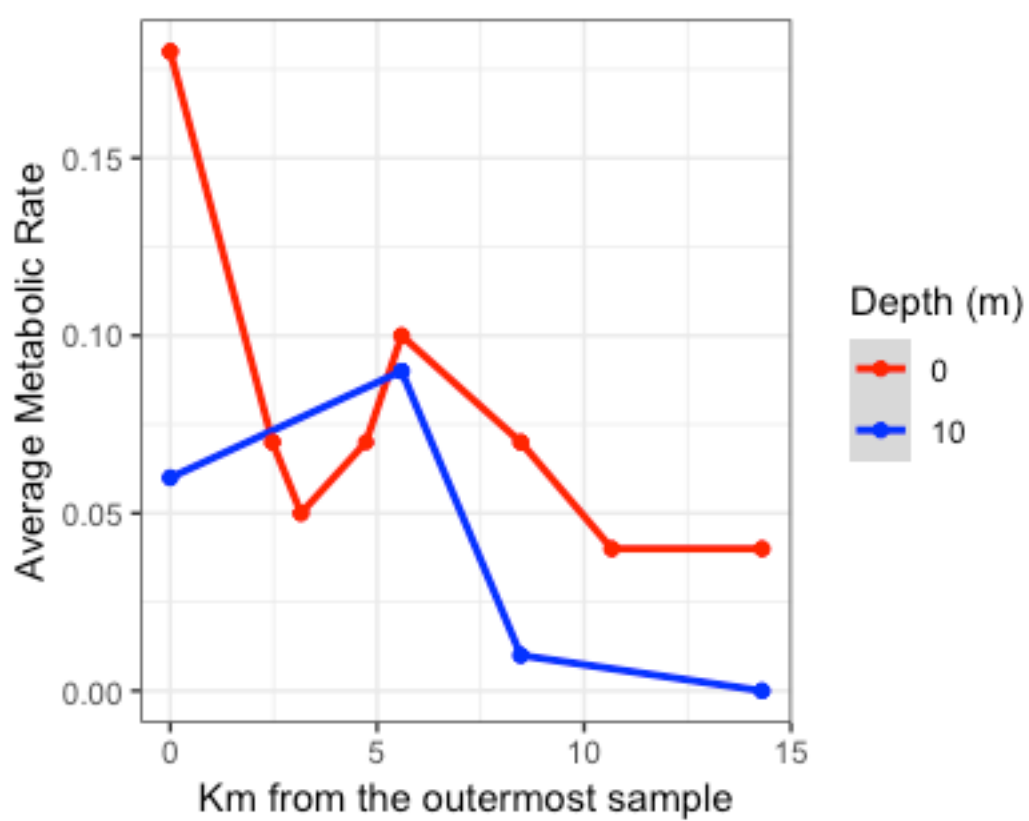

Supplement: Supplementary file 5 — Supplementary Figure S4. [file 41598_2021_98519_MOESM5_ESM.pdf]

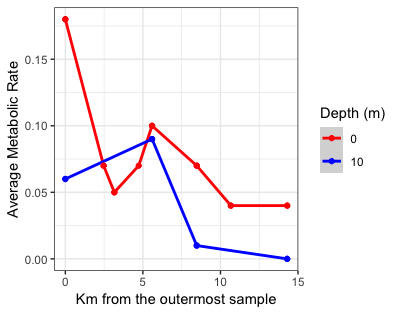

Supplement: Supplementary file 6 — Supplementary Figure S5. [file 41598_2021_98519_MOESM6_ESM.tiff]
