# Supplementary material for: Changes in microbial community phylogeny and metabolic activity along the water column uncouple at near sediment aphotic layers in fjords
Source: Sci Rep. 2021 Sep 29;11:19303. doi: 10.1038/s41598-021-98519-2 (PMC8481465; doi:10.1038/s41598-021-98519-2)
Supplement: Supplementary file 7 — Supplementary Table S1. [file 41598_2021_98519_MOESM7_ESM.pdf]

Data type	Assessing:	Test	Strength	p-value	Significance level
Five fjords	16S Community	anosim	0.248	0.002	**
Five fjords	16S Community	adonis	0.226	0.001	***
Five fjords	Biolog	anosim	0.108	0.039	*
Five fjords	Biolog	adonis	0.261	0.017	*
Horizontal	16S Community	anosim	0.566	0.001	***
Horizontal	18S Community	anosim	0.651	0.001	***
Horizontal	16S Community	adonis	0.24	0.001	***
Horizontal	18S Community	adonis	0.192	0.001	***
Horizontal	Biolog	anosim	-0.05	0.676	
Horizontal	Biolog	adonis	0.286	0.019	*
Vertical	16S Community	anosim	0.728	0.001	***
Vertical	18S Community	anosim	0.944	0.001	***
Vertical	16S Community	adonis	0.28	0.03	*
Vertical	18S Community	adonis	0.153	0.012	*
Vertical	Biolog	anosim	1	0.001	***
Vertical	Biolog	adonis	0.459	0.014	*
